# The Alleged Inhumanity at Guy's

**Published:** 1887-03-26

**Authors:** G. W. Potter


### The Alleged Inhumanity at Guy's.

By G. W. Potter, M.D.

Will you give me space to relate an incident of my own experience, which bears upon the case of the man who was turned out of Guy's Hospital with a broken leg? About three years ago I was sent for to the house of Captain W., in —— Place. When I arrived the Captain was sitting in the dining-room, apparently quite well. He said that Mrs. W. had sent for me to see him, but he did not think he needed my services at all. I inquired what was the matter, and was more than a little surprised when he replied that "he had only got a broken leg." He told me he had fallen, I think on the deck of a steamer, at Hamburg, three days before, and had broken the small bone of his leg. A German surgeon had put it up for him in a plaster-of-paris bandage, and had allowed him to start for England immediately. He had walked about on the deck of the steamer during the voyage, and, as he said, "intended to go out about his usual avocations next day." I took off the bandage and found the fibula fractured a little above the ankle joint. Finding no swelling or other complication, I applied a fresh plaster-of-paris bandage, and gave him permission to go out as he desired. He kept on the bandage for several weeks, walking about every day, and ultimately made a perfect recovery without a bad symptom.

It is not necessary to explain to professional readers the reason for this apparent simplicity of treatment in such a case. But your unprofessional readers ought to be informed that the small bone of the leg is merely a kind of addendum to the large bone, for the purpose of facilitating movement. When simply fractured it can be fixed with the most complete immovability between the large bone and the plaster-of-paris bandage. The foot is at the same time immovably fixed, a bandage suspended from the shoulders slings the foot, and then with crutches there is no risk whatever. The patient will retain his general health and recover the use of his limb much better by going about in the open air than he would by lying in bed. In the case of Captain W., he utterly derided the use of the sling or crutches.

A word more. I have been connected with hospitals, in various capacities, for nearly twenty years, and never once saw the least indication of anything but the most perfect kindness and consideration for any patient during all that time. In general hospitals unkindness and unskilful management are practically impossible.

